# Aged mesenchymal stem cells and inflammation: from pathology to potential therapeutic strategies

**DOI:** 10.1186/s13062-023-00394-6

**Published:** 2023-07-18

**Authors:** Xue Yang, Ying Wang, Valentina Rovella, Eleonora Candi, Wei Jia, Francesca Bernassola, Pierluigi Bove, Mauro Piacentini, Manuel Scimeca, Giuseppe Sica, Giuseppe Tisone, Alessandro Mauriello, Lixin Wei, Gerry Melino, Yufang Shi

**Affiliations:** 1 Department of Experimental Medicine, TOR, University of Rome Tor Vergata, Rome, 00133 Italy; 2 The Third Affiliated Hospital of Soochow University, Institutes for Translational Medicine, State Key Laboratory of Radiation Medicine and Protection, Medical College of Soochow University, Suzhou, Jiangsu China; 3 Center for Translational Medicine, Shanghai Key Laboratory of Diabetes Mellitus, Shanghai Sixth People’s Hospital, Shanghai Jiao Tong University School of Medicine, Shanghai, 200233 China; 4 School of Chinese Medicine, Hong Kong Baptist University, Kowloon Tong, Hong Kong China; 5 Department of Tumor Immunology and Gene Therapy Center, Third Affiliated Hospital of Naval Medical University, Shanghai, 200438 China

**Keywords:** Mesenchymal stem cells, Inflammation, MSC Therapy

## Abstract

Natural ageing of organisms and corresponding age-related diseases result mainly from stem cell ageing and “inflammaging”. Mesenchymal stem cells (MSCs) exhibit very high immune-regulating capacity and are promising candidates for immune-related disease treatment. However, the effect of MSC application is not satisfactory for some patients, especially in elderly individuals. With ageing, MSCs undergo many changes, including altered cell population reduction and differentiation ability, reduced migratory and homing capacity and, most important, defective immunosuppression. It is necessary to explore the relationship between the “inflammaging” and aged MSCs to prevent age-related diseases and increase the therapeutic effects of MSCs. In this review, we discuss changes in naturally ageing MSCs mainly from an inflammation perspective and propose some ideas for rejuvenating aged MSCs in future treatments.

## Background

Ageing greatly increases the risk for many chronic diseases that profoundly threaten the health of elderly individuals [1]. Most age-related diseases, such as atherosclerosis, nonalcoholic steatohepatitis (NASH), osteoarthritis, Alzheimer’s disease (AD), cardiovascular disorders and several cancers [2–8], result from continuous low-level inflammation called “inflammaging” [9]. Normal levels of inflammation can help organisms defend against microbial invasion and repair tissues, and the inflammatory response is attenuated when it is no longer needed [10]. In aged organisms, inflammation appears to be difficult to control, and therefore, inflammatory responses persists at low levels. Multiple mechanisms have been reported to contribute to age-related inflammation, including redox stress responses, glycation, mitochondria dysfunction, and immune system deregulation [11]. However, altered negative regulation of inflammation deserves attention.

Ageing of stem cells (SCs) is involved in age-related diseases. MSCs are endowed with a potent immune suppression capacity in the inflammatory environment due to their ability to inhibit T, B, dendritic, and natural killer cell functions and to favour macrophage polarization no acquisition of an anti-inflammatory phenotype [12]. Gene expression profiling of bone marrow-derived MSCs isolated from the femoral heads of elderly and middle-aged donors revealed differentially expressed genes in pathways related to ageing, such as oxidative stress-induced DNA damage, telomere attrition, differentiation and epigenetic regulatory pathways [13–17]. Here, we focus on alterations to aged MSCs that are associated with inflammaging.

Cell therapy based on MSCs [18–23] is a promising treatment for many diseases, especially immune-related diseases [24–26]. However, the effects of MSC therapy are not always satisfactory in some elderly patients, such as those who undergo autologous transplantation [27–30]. This observation indicates that ageing probably affects the therapeutic efficacy of MSCs. Quality control of aged MSCs and improves to the ageing microenvironment of patients are probably both important strategies for realizing the highest therapeutic efficacy. Ageing has indeed been demonstrated to affect various functions of MSCs. Thus, development of strategies to increase the therapeutic effect of aged MSCs is an urgent need. Furthermore, the undesirable role of senescent cells can be minimized, even in cases in which young MSCs are employed. In this review, we discuss changes to naturally ageing MSCs and how these alterations contribute to inflammaging. Furthermore, we provide perspective on ways to increase the therapeutic effects of aged MSCs.

### Characteristics of aged MSCs

**Fig. 1:**
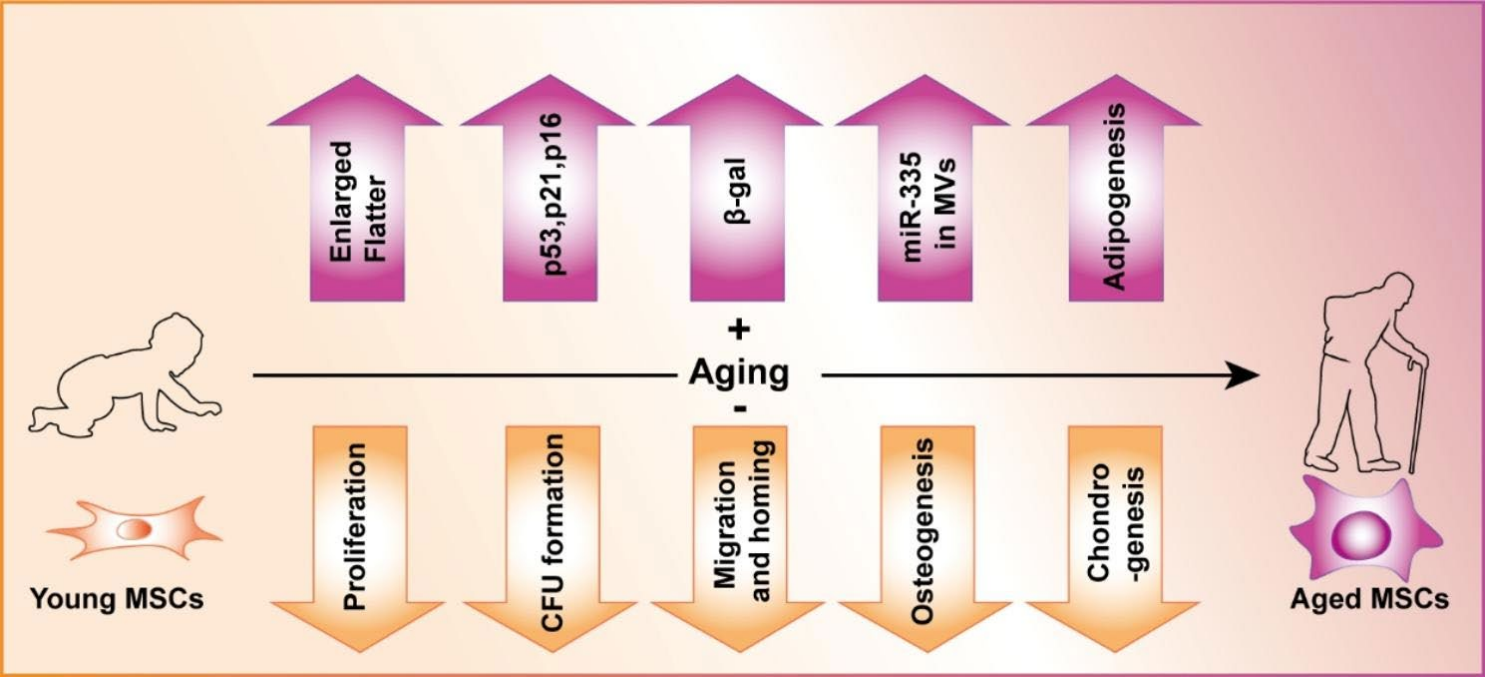
Characteristics of aged MSCs With ageing, MSCs express many senescence markers, including an enlarged and flattened cell shape, upregulation of p53, p21, and p16 expression, increased activation of beta-galactosidase, and increased miR-335 expression in MVs. Ageing suppressed some basic functions of MSCs, including their proliferation, CFU formation, migration and homing. Aged MSCs also present a skewed trilineage differentiation pattern, with enhanced adipogenesis and reduced osteogenesis and chondrogenesis rates

In studies of aged MSCs, senescence and ageing are often conflated, but these processes are very different. Generally, senescence is a cellular programme mainly activated by stress, including stress caused by DNA damage, oncogene activation, and replication-related telomere shortening [31] both in vivo and in vitro. Ageing is a natural condition of individuals caused by the passage of time [32]. Thus, aged MSCs are chronologically ageing cells that are retained in old organisms (in humans, older than 60 years and, in transgenic mice, 18 months old or older) (Fig. 1).

#### Senescent phenotype and impaired contribution to the stem cell pool

To some extent, aged MSCs share some common features with senescent cells [33]. Both present with an enlarged, flat morphology instead of a spindle shape. Similar to those in senescent cells, the activation of the p53 pathway and the expression of its target genes, p21 and p16^Ink4α^, are commonly used as markers, in addition to the increased activation of β-galactosidase, to distinguish aged MSCs [34, 35]. During ageing, the number of MSCs in vivo decreases, and this outcome is probably due to a decline in proliferative and cell colony-formation ability [36, 37]. These are among the reasons why autologous MSC therapy in ageing patients is hampered [38], and stem cell exhaustion has been suggested to be a hallmark of ageing [39, 40]. In parallel to the inhibited-proliferation phenotype, the expression of genes involved in apoptosis and cell cycle inhibition, including p53, p21, p16^Ink4α^ and Bax, is upregulated in bone marrow MSCs isolated from 23- to 24-month-old mice [41]. Mesenchyme homeobox 2 (MEOX2), which functions as a negative regulator of proliferation, has been found to be upregulated in aged MSCs [42]. Extracellular microvesicles have been proposed to be novel biomarkers of ageing MSCs [43]. For instance, the expression of miR-335 in extracellular microvesicles has been found to correlate with the age of donors of human MSCs, and overexpression of miR-335 caused the acquisition of a senescence phenotype and reduced the differentiation potential of MSCs [44].

#### Skewed differentiation

Regarding the effect of ageing on the differentiation potential of MSCs, particular attention has been given to adipogenesis, osteogenesis and chondrogenesis. Decreased osteogenic and chondrogenic differentiation potential in aged MSCs has been reported in several studies [37, 45–47]. In addition, during MSC ageing, adipogenesis is favoured, while osteogenesis is simultaneously disfavoured, impairing bone formation capacity [48–50] and subsequent osteoporosis [48, 51, 52]. These findings are consistent with the effects of increased adipose tissue accumulation in bone marrow during ageing. This shift in cell differentiation programmes is likely due to changes in gene expression. For instance, ageing triggers changes in the transcription of the nuclear receptor peroxisome proliferator-activated receptor gamma (PPARγ) and CCAAT/enhancer binding protein alpha [C/EBPα] and inhibits the expression of Runt-related transcription factor2 (RUNX2) [53, 54].

In contrast, a study by Khanh and colleagues reported that the brown/beige adipocyte differentiation capacity of adipose MSCs derived from young and aged donors was inversely correlated with age. Impaired expression of Sirt1 was shown to be critical for repressed beige adipocyte differentiation [55]. These data, which seem contradictory, suggested that the effect of ageing on adipocyte differentiation ability is probably dependent on the specific type of adipocyte involved. In addition, MSCs constitute a population of adult stem cells with obvious heterogeneity, which means that analyses of MSCs from different subgroups and different donors might lead to different results.

In addition to the altered differentiation potential governed by gene expression changes during ageing, MSCs also show the potential to transdifferentiate into endothelial-like cells in both in vivo and in vitro systems [56–58]. Duscher et al. discovered age-related depletion of a pro-vascular subpopulation of MSCs via single-cell transcription analysis, and these results indicated the reduced ability of aged MSCs to support vessel formation [59].

#### Decreased migration and homing properties

Migration and homing to injury and inflammatory sites are necessary for MSCs to facilitate tissue injury repair and immunosuppression. The release of signalling factors such as cytokines and chemokines results in MSC recruitment. Stromal derived factor-1 [SDF-1] is one of the most important MSC-recruiting chemokines secreted at an injury site, and this effect is mediated by SDF-1 binding to its receptor, C-X-C motif chemokine receptor 4 (CXCR4), which is expressed on the surface of MSCs. In addition, various other chemokine receptors, such as C-C chemokine receptor type 2 (CCR2), C-C chemokine receptor type 27 (CCR7), C-X-C motif chemokine receptor 5 (CXCR5), and C-X3-C Motif Chemokine Receptor 1 (CX3CR1) [60–63], are involved in MSC migration. Proteinases such as matrix metalloproteinases (MMPs) are also crucial for degrading components of the extracellular matrix and generating space to allow MSC migration [64–67]. Several studies have reported that aged MSCs display a lower ability to home to wound sites relative to the ability of young MSCs [36, 68]. Moreover, by measuring the migration rate in vitro, Geibler et al. showed that young MSCs showed significantly higher migratory potential than aged MSCs throughout several passages in culture [69]. Consistent with these observations, the expression of CXCR4 and C-X-C motif chemokine receptor 7 (CXCR7) on the surface of aged MSCs has been found to be significantly reduced compared to that in young counterparts [70, 71]. Tumour necrosis factor receptor (TNFR), interferon-γ receptor (IFNGR) and CCR7 level reduction was also involved in the age-dependent decrease in the migratory capacity and the activation of bone marrow MSCs [68]. Impaired migration of aged MSCs suggests an attenuated response of aged MSCs to injury signals.

### Altered properties dominated by p53

p53 has attracted much attention in the context of MSC ageing because of its crucial role in cell cycle arrest and regulated expression of other age-related genes. In addition, it has been demonstrated that p53 knock-in mice showed obvious signs of organismal ageing [72–74].

#### Role of p53 in the osteogenesis of aged MSCs

As a transcription factor, p53 not only regulates the cell cycle [75–78] but is also involved in bone formation [79–81]. Upregulation of p53 pathway activity in the context of ageing may play a critical role in mediating the reduction in osteoblastogenesis by human MSCs, indicating that intrinsic alterations in human MSCs with ageing may contribute to skeletal ageing in humans [81]. Osteosclerosis has been detected in p53-knockout mice, and there is also evidence suggesting that p53 regulates osteoblast differentiation through the action of the transcription factors Runx2 and Osterix [82]. p53 induces the transcription of several miRNAs, including miR-29 and miR-34a, to regulate stemness and differentiation [83, 84]. Another study identified an additional miRNA, miR-145a, targeted by p53. The authors found that the p53/miR-145a/Cbfb axis inhibited the osteogenic differentiation of MSCs [80]. Overexpression of p53 inhibited osteogenesis in young MSCs in culture and in those implanted into NOD/SCID mice by inhibiting the transcription of the miR-17‐92 cluster, which is decreased in aged mice. More importantly, Smurf, a direct target gene of miR‐17, plays an important role in the p53/miR‐17 cascade during osteogenesis [85].

#### Preventive role of p53 in the tumor cell transformation of aged MSCs

As a cancer suppressor gene [86, 87], p53 plays a key role in the prevention of the tumoral cell transformation of MSCs. Fibrosarcomas frequently develop in aged mice. Hanchen et al. used a genetically tagged bone marrow (BM) transplantation model to show that aged mice develop MSC-derived fibrosarcomas. They also showed that transplantation of aged MSCs recapitulated the development of naturally occurring fibrosarcomas in old mice, with gene expression changes or p53 mutations similar to those identified in an in vivo model [88]. A study verified the effect of p53 on the tumoral transformation of AT-MSCs by using p53-knockout mice. The authors showed that wild-type or p21^−/−^p53^+/+^ MSCs did not show any sign of tumor cell transformation of the MSCs. However, loss of p53 favoured the fibrosarcoma formation by MSCs after either subcutaneous or intrafemoral injection in immunodeficient mice [89]. Inactivation of Rb and p53 in BM-MSC-derived osteogenic progenitors has been proven to give rise to osteosarcoma-like tumours [90].

Mechanistically, p53 mutations drive the tumoral transformation of aged MSCs, which is probably dependent on the loss of p53 binding to the survivin gene promoter, leading to abnormally upregulated survivin expression, which ultimately results in unlimited cell proliferation [91]. The mutation of p53 in aged mice is the main reason for the tumoral transformation of aged MSCs. Thus, upregulation of p53 in aged MSCs may be an underlying mechanism of inherent self-protection and homeostatic maintenance.

### Involvement of Aged MSCs in inflammaging

Inflammaging has been described as the persistence of long-term and low-grade systemic chronic inflammation [92]. For two decades, inflammaging has been widely studied and has emerged as an important concept to enable dynamic reassessments of the immune responses in elderly people [93, 94]. Inflammaging is a strong risk factor for many chronic diseases in elderly individuals. Unbalanced inflammation can lead to severe organ damage and disrupt homeostasis. Multiple mechanisms have been reported to contribute to inflammaging [11], including redox stress, glycation, dysfunction of mitochondria, and deregulation of the immune system [95–97]. All of these mechanisms are related to accelerated inflammation. Increasing evidence indicates that aged MSCs can also contribute to inflammaging.

#### Activation of the innate immune system

The accumulation of damaged macromolecules and cellular debris can trigger inflammaging because both conditions activate innate immunity via damage-associated molecular patterns (DAMPs) [98, 99]. The inflammaging process involves macrophages that secrete high amounts of proinflammatory cytokines and chemokines [100]. These factors, in turn, activate inflammatory signalling pathways, including the NF-κB and STAT pathways [101, 102].

**Fig. 2:**
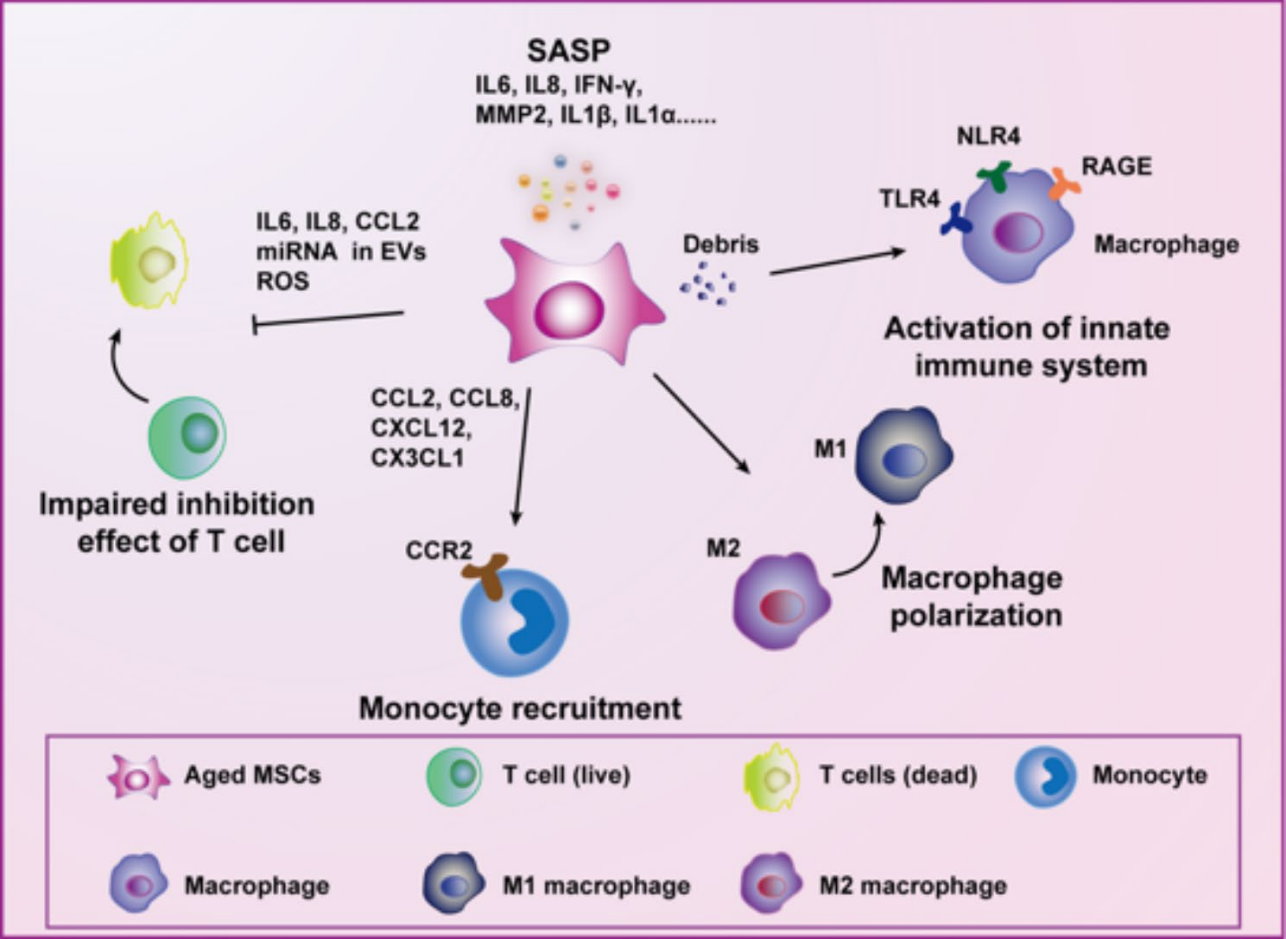
Involvement of aged MSCs in the development of inflammaging. Ageing MSCs secrete a large amount of SASP factors, favouring the formation of an inflammatory microenvironment. Debris produced in aged MSCs can be considered a kind of DAMP and induces macrophage activation through receptors expressed on these cells; for example,, TLR4, NLR4 and RAGE. Aged MSCs promote monocyte recruitment by secreting ligands of CCR2 and induce the shift of macrophages from the M2 to the M1 phenotype. The immunosuppressive capacity of MSCs decreases with ageing and manifests by the reduced inhibitory effects of T cells mediated through IL-6, IL-8, CCL2, and miRNA carried by EVs and ROS production

The debris of aged MSCs are relevant sources of DAMPs. During ageing, MSCs are enlarged and heterogeneously shaped and granules and cell inclusions accumulate in the cytoplasm, forming cellular debris [103]. Debris from MSCs can induce innate immune responses through the activation of innate immune cell receptors, including NOD-like receptor 4 (NLR4) and Toll-like receptor 4 (TLR4) [104–107]. Chemokines and cytokines secreted by cells in the activated innate immune system further promote inflammation cascade activation that may contribute to inflammaging (Fig. 2).

#### Secretion of inflammatory factors by MSCs with SASP 

Aged MSCs share similar phenotypical traits with senescent cells, including the senescence-associated secreted phenotype (SASP), which characterizes high levels of secreted inflammatory bioactive factors, including cytokines, chemokines and proteinases. The SASP plays an essential role in mediating MSC conversion from an anti-inflammatory cell type to a proinflammatory cell type. A significant increase in the expression levels of several SASP molecules, such as monocyte chemoattractant protein-1 (MCP-1), interleukin 6 (IL6), interleukin 8 (IL8), interleukin 1β (IL1β) and interleukin 1α (IL1α), have been revealed in aged MSCs compared to young MSCs (Fig. 2). Protein analysis of conditioned medium obtained from cultures of young and aged MSCs showed a marked increase in the secretion of MCP-1, IL6, IL8, IL1*α*, C-X-C motif ligand 2 (CXCL2) and C-C motif ligand 4 (CCL4) in aged MSCs relative to young MSCs [108]. Other studies have also reported that aged MSCs release excessive secretome factors, including IL6, IL8, IFN-γ, MCP-1, C-C motif ligand 12 (CCL12), C-C motif ligand 11 (CCL11), WNT1-inducible-signalling pathway protein 1 (WISP-1) and matrix metalloproteinases (e.g., MMP2) [109–111]. IL6 is considered the most important cytokine in inflammaging. Augmented IL6 expression in elderly people has been associated with reduced physical abilities, cognitive dysfunction, the onset of cancers, and disease progression of general degenerative disorders [111].

The SASP of aged MSCs is probably related to the persistent increase in the activation of TLR signalling [112]. Enhanced adipogenesis with ageing might contribute to acquisition of the SASP [113, 114]. Exosomes, which are secreted cellular microvesicles, also play important roles in the inflammatory secretome of MSCs [115].

#### Aged MSCs facilitate monocyte recruitment and a proinflammatory macrophage polarization shift

Martini and colleagues reported that cardiac MSCs acquire the ability to express chemokines, such as *CCL2, CCL8, CXCL12*, and *CX3CL1*, which are ligands of the CCR2 receptor and play major roles in monocyte recruitment. By using an inhibitor of CCR2, the authors proved that aged cMSCs promoted monocyte recruitment through the action of CCR2, thus contributing to inflammaging [116].

MSCs are able to promote the polarization of macrophages from the M1 to the M2 phenotype to exert anti-inflammatory effects [117]. However, SASP factors have been demonstrated to polarize macrophages by shifting the M2 phenotype towards the M1 phenotype [118] (Fig. 2). Thus, aged MSCs affect macrophage polarization in opposite ways [119]. Interestingly, although macrophages cocultured with young MSCs expressed M2 phenotype markers, such as arginase 1 (Arg1) and interleukin 10 (IL10), those cocultured with aged MSCs showed increased expression of M1 phenotype-related tumour necrosis factor-α (TNF-α). In addition, macrophages cocultured with aged MSCs exhibited increased migratory ability, which is a property typical of classically activated M1 macrophages [120]. As described above, aged MSCs can produce essential inducers of M1 macrophage differentiation, including IFN-γ and IL1, and activate the NF-κB signalling pathway [121].

#### Impaired inhibition effect of T cells

The immunosuppressive effects of MSCs are mainly directed towards T cells. However, aged MSCs display a diminished capacity to suppress T cell proliferation and activation [122, 123]. Allogeneic coculture systems show that young MSCs were able to effectively inhibit phytohemagglutinin (PHA)-induced PBMC proliferation, while aged MSCs exhibited a significantly reduced ability to impair PHA-PBMC proliferation [109]. Another study reported that the suppressive ability of aged MSCs on both CD4^+^ and CD8^+^ T-cell proliferation was impaired compared with that of young MSCs. The diminished ability to suppress T cells is probably due to SASP factor secretion, since neutralization of IL6, IL8 and CCL2 enhances the immunomodulatory function of elderly MSCs [123]. Priming MSCs with proinflammatory factors stimulates the production of extracellular vesicles (EVs), which exhibit potent anti-inflammatory effects and enhanced therapeutic potential [124, 125]. Alteration of EVs and related miRNAs may explain the impaired immunomodulation properties of aged MSCs. EVs secreted by MSCs obtained from an aged donor showed reduced immunosuppression activity relative to MSC-EVs obtained from a younger donor [126]. These differences were ascribed to ectopic levels of MSC-EV miRNAs, including miR-223-5p, miR-125b-5p and miR-127-3p. These data support previous findings highlighting that age-related alterations in miRNA amounts in MSCs-EVs were associated with altered immunomodulatory properties of aged MSCs [127–129]. AT-MSCs obtained from elderly people were characterized by increased oxidative stress compared to MSCs obtained from younger people, and ROS decreased the ability of MSCs to suppress T cells [130].

A series of immunosuppressive molecules, such as indoleamine-2,3-dioxygenase (IDO) (in humans), inducible nitric oxide synthase (iNOS) (in rodents), prostaglandin E2 (PGE2), transforming growth factor β (TGF-β), tumour necrosis factor-inducible gene 6 protein (TSG6), and IL10, have been demonstrated to exert immunosuppressive effects on MSCs [131]. In particular, IDO has been found to be downregulated in replication-associated senescent MSCs [132, 133]. However, there is an urgent need to expand this research to identify the mechanisms underlying the decreased immunosuppression of naturally aged MSCs.

### Potential strategies to rejuvenate aged MSCs

**Fig. 3:**
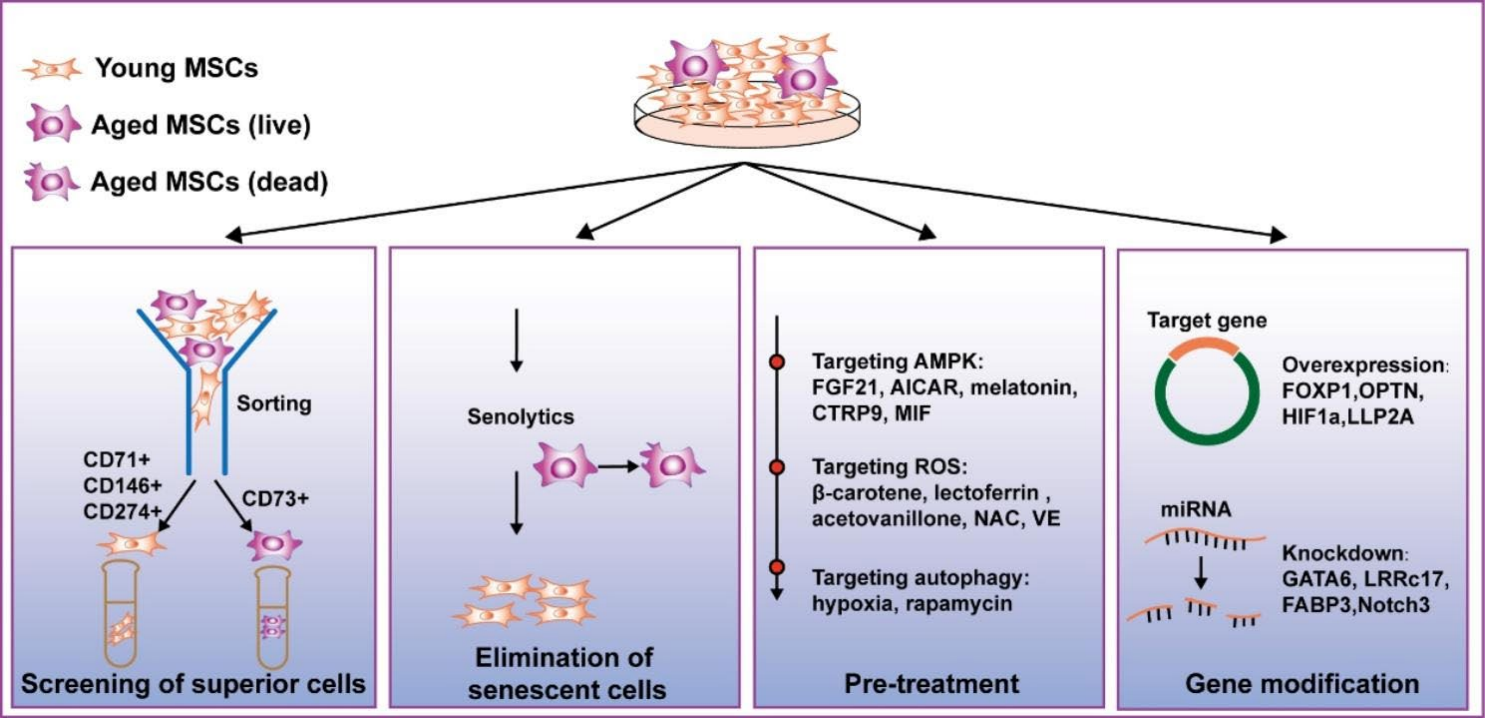
Strategies to rejuvenate aged MSCs Aged MSCs suitable for treatment can be obtained by sorting surface markers. Senescent MSCs can be deleted with senolytics. Pretreatment can be performed with several reagents able to target the AMPK signalling pathway, ROS and autophagy factors to rejuvenate aged MSCs. Gene modification for inducing the upregulation or knocking down some target genes favours aged MSC rejuvenation

MSCs have been widely used for treating immune-related diseases, and their immunosuppressive capacity has been evaluated in clinical trials. More than 12% of clinical trials of MSC-based treatments have been performed for immune-related diseases, such as graft-versus-host disease (GvHD), Crohn’s disease, psoriasis, urticaria, and arthritis (https://www.clinicaltrials.gov/). However, the therapeutic effects have been unsatisfactory, probably due to the quality of the MSCs. Ageing is an important factor affecting the therapeutic effects of MSCs, especially for use in autologous transplantation in elderly individuals. Thus, determining how to improve the therapeutic efficacy of aged MSCs is an urgent need for promoting the clinical applications of MSCs (Fig. 3).

#### Identifying superior cells

Based on many studies, the heterogeneity of MSCs increases with ageing. Lise Lefèvre et al. identified a CD73^+^ kidney mesenchymal stromal cell (kMSC) population that was increased in the kidneys of ageing organisms. Aged CD73^+^ kMSCs displayed senescence-associated hallmarks, including a low proliferation rate and DNA damage accumulation. In addition, these cells produced niche factors required to recruit monocytes, ultimately promoting a positive regulatory loop in response to local inflammation [134]. Another study showed that CD271^+^CD146^+^ MSCs were predominant in children, while CD271^+^CD146^−^ MSCs were most common in adults [135], with the number of CD271^+^ cells declining with increasing donor age [136]. CD71^+^, CD146^+^ and CD274^+^ BM-MSCs are reported to be negatively correlated with donor age [137]. Based on the heterogeneity of aged MSCs, depletion of a subgroup of aged MSCs or enrichment of a subgroup with young features maintained in vitro before transplantation may be alternative cell to increase the therapeutic effect of aged MSCs.

#### Elimination of senescent cells

Eliminating senescent cells delays the onset of several pathologies and allegedly promotes a healthy lifestyle and increased lifespan [138, 139]. Many studies have identified agents that specifically eliminate senescent cells through the production of senolytic compounds [140]. Most of these products function mainly through apoptosis induction and immune system activation to eliminate senescent cells. Senolytics can be categorized into several classes according to their different targets; they include the B-cell lymphoma 2 (BCL-2) family, HSP90 and the p53 pathway compounds [141]. Some senolytic effects are produced via the combination of two drugs, such as dasatinib plus quercetin (D + Q), which have been proven to remove naturally occurring senescent cells from human adipose tissue [142]. D + Q has also been evaluated for use in treating age-associated diseases in clinical trials (https://www.clinicaltrials.gov/), and D + Q allegedly showed significant beneficial effects [143], indicating it exhibits some potential as a combination senolytic agent.

#### Pretreatment

Several studies have indicated that aged MSCs can be rejuvenated. MSCs derived from aged donors showed a lower level of fibroblast growth factor 21 (FGF21), and overexpression of FGF21 in aged MSCs inhibited senescence via the AMP-activated protein kinase (AMPK) signalling pathway. Thus, targeting FGF21 might represent a novel strategy to increase the quality of aged MSCs [144]. The expression of miR-155-5p was much higher in MSCs obtained from aged donors than in MSCs obtained from young donors, and miR-155-5p downregulation decreases senescence of aged MSCs by activating AMPK signalling. Hence, an AMPK activator, AICAR, may be used to renew aged MSCs [145]. Other AMPK activators, such as melatonin [146], C1q/tumour necrosis factor-related protein 9 (CTRP9) [147], and macrophage migration inhibitory factor (MIF) [148], have also been reported to exert an antiaging effect on MSCs.

ROS production is the major contributor to MSC ageing and age-related diseases. Compounds with antioxidant activity, such as lactoferrin [149], acetovanillone, N-acetyl cysteine (NAC), NAC and l-ascorbic acid 2-phosphate, vitamin E, metformin, fullerol, fucoidan, carvedilol, nicorandil and 5-azacytidine, might be able to rejuvenate aged MSCs [150]. β-Carotene is another candidate for rejuvenation of aged MSCs. It has been reported that β-carotene can relieve ageing in MSCs, as evidenced by the reduced expression of p16 and p21. β-Carotene has also been shown to reduce ageing rates in tissues and organs in vivo and appeared to inhibit ageing caused by antioxidative stress by regulating KAT7-P15 signalling [151].

Hypoxia has been demonstrated to suppress the senescence of aged MSCs in several reports. Hypoxic preconditioning enhanced the in vivo angiogenic capacities of human AT-MSCs obtained from older donors. Hypoxia also repressed the expression of ageing-associated gene p16^Ink4α^ and ageing inducer aminoacyl-tRNA synthetase‐interacting multifunctional protein 3 (AIMP3) [152]. Hypoxia probably reverses the ageing of MSCs partially by activating autophagy [153]. Rapamycin, an autophagy activator that inhibits the mammalian target of rapamycin (mTOR) pathway, has also been reported to exert an antiaging effect on MSCs and to enhance the immunomodulatory potency of MSCs [154]. Interestingly, another study reported that young MSC-derived apoptotic vesicles were able to restore nuclear alterations, as well as the self-renewal and osteogenic and adipogenic lineage differentiation capacity of aged bone marrow MSCs via autophagy activation [155].

#### Gene modifications

In addition to pretreatment, gene modification is a widely used method to modify MSCs. There have only been a few studies using gene modification as a strategy to rejuvenate MSCs and recover their function. In a study of MSC reprogramming, which was similar to an approach used for cell rejuvenation, the expression of GATA-binding protein 6 (GATA6) was found to be attenuated, and that of Forkhead box P1 (FOXP1) was upregulated. Thus, knocking down GATA6 and inducing FOXP1 overexpression showed the potential to ameliorate the expression of cellular hallmarks of ageing [156]. Liu et al. found that leucine-rich repeat-containing 17 (LRRc17) expression in BM-MSCs was highly positively correlated with age. LRRc17 knockdown rejuvenated aged MSCs and increased their therapeutic efficacy in the context of osteoporosis [157]. In another study on MSC treatment in OP, it was reported that reactivating optineurin or inhibiting FABP3 activity rescued osteoporotic phenotypes [49]. AIMP3-induced senescence was negatively regulated by hypoxia‐inducible factor 1α (HIF1α) and positively regulated by Notch3, which means that HIF1α overexpression and Notch3 knockdown inhibited the senescence of MSCs [152].

## Conclusions

MSCs undergo numerous changes with natural ageing, including acquisition of a senescence phenotype and reductions in differentiation, migratory and homing ability, and they contribute to inflammaging in the ageing body. All these alterations are probably due to changes at the molecular level, including gene expression changes and epigenetic modifications [33]. These changes support the view that stem cell ageing results in body ageing and age-related diseases. Therefore, aged MSCs can be considered targets for therapy developed to prevent or attenuated inflammaging. A considerable number of senolytics have been identified for targeting and eliminating senescent cells in vivo.

The changes in MSC property with ageing not only favour inflammaging but also affect their therapeutic potential. Indeed, impaired osteogenic differentiation, migration or immune regulation reduces the therapeutic effect of MSCs in tissue injury repair and inflammation-related disease treatment. The undesirable effects of senescence in aged MSCs can be minimized in several ways, as previously discussed. The same approaches might also be employed to increase therapeutic effects, even on young MSCs. Notably, a study on human gingival tissue MSCs (GMSCs) reported that irrespective of donor age, GMSCs displayed effective neurogenesis, immunoregulation and regenerative potential [158–161]. These findings suggest a possible option for treating elderly patients who need autologous transplantation of MSCs. However, MSC therapy was less effective in an elderly cohort than in a young cohort, indicating that old plasma might carry factors that inhibit the function of MSCs [162]. Murphy and coauthors proposed that growth differentiation factor 11 (GDF11), mTOR, CCL11, and the insulin/insulin-like growth 1 (IGF1) signalling pathways may be relevant to stem cell function [163, 164]. Therefore, in addition to MSC quality, the microenvironment of patients also needs to be considered or improved for successful MSC therapy.

Based on the mechanisms underlying MSC function that are affected by ageing, the development of develop novel strategies to ameliorate inflammaging and to increase the therapeutic efficacy of aged MSCs is promising.
